# A *n*-3 PUFA depletion applied to rainbow trout fry (*Oncorhynchus mykiss*) does not modulate its subsequent lipid bioconversion capacity

**DOI:** 10.1017/S0007114516004487

**Published:** 2017-01-23

**Authors:** Julie Mellery, Jonathan Brel, Junio Dort, Florian Geay, Patrick Kestemont, David S. Francis, Yvan Larondelle, Xavier Rollin

**Affiliations:** 1 Institut des Sciences de la Vie, Université catholique de Louvain, Croix du Sud, 2/L7.05.08, 1348 Louvain-la-Neuve, Belgium; 2 Unité de Recherche en Biologie Environnementale et Evolutive, Université de Namur, Rue de Bruxelles, 61, 5000 Namur, Belgium; 3 School of Life and Environmental Sciences, Deakin University, PO Box 423, Warrnambool, VIC 3280, Australia

**Keywords:** Rainbow trout, Fatty acid metabolism, Lipid bioconversion capacity, Plant-derived oils, Whole body fatty acid balance method

## Abstract

Nutritional strategies are currently developed to produce farmed fish rich in *n*-3 long-chain PUFA (LC-PUFA) whilst replacing fish oil by plant-derived oils in aquafeeds. The optimisation of such strategies requires a thorough understanding of fish lipid metabolism and its nutritional modulation. The present study evaluated the fatty acid bioconversion capacity of rainbow trout (*Oncorhynchus mykiss*) fry previously depleted in *n*-3 PUFA through a 60-d pre-experimental feeding period with a sunflower oil-based diet (SO) followed by a 36-d experimental period during which fish were fed either a linseed oil-based diet (LO) (this treatment being called SO/LO) or a fish oil-based diet (FO) (this treatment being called SO/FO). These treatments were compared with fish continuously fed on SO, LO or FO for 96 d. At the end of the 36-d experimental period, SO/LO and SO/FO fish recovered >80 % of the *n*-3 LC-PUFA reported for LO and FO fish, respectively. Fish fed on LO showed high apparent *in vivo* elongation and desaturation activities along the *n*-3 biosynthesis pathway. However, at the end of the experimental period, no impact of the fish *n*-3 PUFA depletion was observed on apparent *in vivo* elongation and desaturation activities of SO/LO fish as compared with LO fish. In contrast, the fish *n*-3 PUFA depletion negatively modulated the *n*-6 PUFA bioconversion capacity of fish in terms of reduced apparent *in vivo* elongation and desaturation activities. The effects were similar after 10 or 36 d of the experimental period, indicating the absence of short-term effects.

There is an expectation on aquaculture to supply fish rich in health promoting *n*-3 long-chain PUFA (*n*-3 LC-PUFA), principally EPA (20 : 5*n*-3) and DHA (22 : 6*n*-3). It is well established that *n*-3 LC-PUFA impart a host of positive effects on human health^(^ ^1^ ^–^ ^3^ ^)^. Moreover, *n*-3 LC-PUFA are essential fatty acids for the optimal growth and health of fish^(^ ^4^ ^,^ ^5^ ^)^. Typically, the high *n*-3 LC-PUFA content in farmed fish is derived from the inclusion of marine-derived fish oil as one of the dietary lipid sources within aquafeeds^(^ ^6^ ^,^ ^7^ ^)^. However, fish oil has become expensive and difficult to source, and given its status as a finite marine resource, its utilisation is widely criticised from a sustainability perspective^(^ ^6^ ^,^ ^7^ ^)^. One of the key sustainable alternatives to fish oil are plant-derived oils^(^ ^8^ ^)^. In contrast to fish oil, plant-derived oils lack LC-PUFA^(^ ^8^ ^)^ but are particularly rich in MUFA and C18 PUFA, especially 18 : 1*n*-9 and linoleic acid (LA, 18 : 2*n*-6), and in certain sources, such as linseed, camelina or perilla oils, rich in *α*-linolenic acid (ALA, 18 : 3*n*-3)^(^ ^5^ ^,^ ^8^ ^)^.

Among fish species, rainbow trout (*Oncorhynchus mykiss*) and other salmonids possess a relatively high capacity to endogenously convert the dietary essential fatty acids LA and ALA into *n*-6 and *n*-3 LC-PUFA through a combination of desaturation steps requiring Δ-6 and Δ-5 desaturases, elongation steps requiring elongases 2 and 5 and partial *β*-oxidation^(^ ^4^ ^,^ ^9^ ^–^ ^13^ ^)^. Previous studies reported increased desaturation and elongation activities without significant detrimental effects to growth and health in salmonids fed on plant-derived oil diets (i.e. sunflower oil^(^ ^14^ ^–^ ^16^ ^)^, olive oil^(^ ^14^ ^)^, palm oil^(^ ^16^ ^)^, rapeseed oil^(^ ^16^ ^,^ ^17^ ^)^ or linseed oil^(^ ^14^ ^,^ ^15^ ^,^ ^17^ ^,^ ^18^ ^)^), as a blend or sole source. In rainbow trout, the complete dietary replacement of fish oil by linseed oil stimulated fatty acid metabolism along the bioconversion pathway^(^ ^18^ ^–^ ^21^ ^)^. However, while providing a highly suitable source of energy for fish growth and maintenance, it is well documented that the fatty acid composition of the dietary lipid source is reflected in fish tissues. Therefore, despite an increase in the bioconversion capacity, fish fed on plant-based diets invariably contained lower EPA and DHA concentrations as compared with those fed on fish oil-based diets^(^ ^15^ ^,^ ^16^ ^,^ ^18^ ^–^ ^21^ ^)^, resulting in major drawbacks from a fish consumption perspective.

There is currently a need to optimise feeding strategies to facilitate the production of farmed fish rich in *n*-3 LC-PUFA whilst minimising fish oil inclusion in aquafeeds. Finishing diets, given before harvest and formulated with fish oil, have been investigated to restore the *n*-3 LC-PUFA content in fish previously fed plant-based diets throughout the grow-out period. Previous studies have demonstrated positive results in many fish species, including Atlantic salmon (*Salmo salar*)^(^ ^22^ ^,^ ^23^ ^)^, common carp (*Cyprinus carpio*)^(^ ^24^ ^)^, red hybrid tilapia (*Oreochromis* sp.)^(^ ^25^ ^)^, European sea bass (*Dicentrarchus labrax*)^(^ ^26^ ^)^, red seabream (*Pagrus auratus*)^(^ ^27^ ^)^ and Murray cod (*Maccullochella peelii peelii*)^(^ ^28^ ^)^. An EPA and DHA recovery rate of approximately 80 % was reported at the end of the finishing period in Atlantic salmon previously fed plant-based diets^(^ ^22^ ^,^ ^23^ ^)^. In rainbow trout, finishing diets induced a shift in fish fatty acid profiles to a more fish oil-like composition, but were unable to achieve similar *n*-3 LC-PUFA concentrations as compared with fish fed on fish oil throughout their growth^(^ ^19^ ^,^ ^29^ ^)^. The efficiency of a finishing period is determined by a combination of factors including the fish species, the finishing period duration, the fatty acid profile of the alternative oil used^(^ ^19^ ^,^ ^25^ ^,^ ^28^ ^)^ (i.e. the dietary C18 PUFA level^(^ ^30^ ^)^) or the application of a short-term feed deprivation period before the commencement of the finishing period^(^ ^29^ ^)^. Although the various feeding strategies that incorporate a finishing strategy demonstrate promising results with undoubtable positive environmental and economic effects, they still rely upon the inclusion of unsustainable dietary fish oil. An alternative strategy involves stimulating fish fatty acid metabolism through nutritional programming during early larval stages as a means of improving the acceptance and conversion of dietary ALA from plant-based diets at juvenile stages^(^ ^31^ ^–^ ^33^ ^)^. Vagner *et al.* ^(^ ^31^ ^)^ observed increased Δ-6 desaturase gene expression in European sea bass juveniles fed an *n*-3 LC-PUFA deficient diet from day 83 post-hatch to day 118, when larvae had been previously fed a low *n*-3 LC-PUFA diet (0·5 % EPA+DHA), as compared with a high *n*-3 LC-PUFA diet (3·7 %). Moreover, in a study where rainbow trout were fed a plant-based diet containing deuterated ALA, a higher conversion of dietary deuterated ALA to DHA was observed in smaller fish (0·5–1·5 g) in comparison to larger fish (6–8 g), highlighting the rapid change of bioconversion capacity with fish size^(^ ^34^ ^)^. Collectively, the results of these studies provide promising insight into the implementation of feeding strategies for the optimisation of EPA and DHA production and retention in fish tissues. However, for the most part these strategies have not been tested in unison, yielding positive yet incremental benefits. To date, the impacts of combined strategies for increasing *n*-3 LC-PUFA deposition currently remain unknown, ultimately requiring dedicated assessment to determine the extent to which utilisation measures can be optimised.

The aim of the present study was to evaluate the fatty acid bioconversion capacity of rainbow trout fry previously depleted in *n*-3 PUFA through feeding on a sunflower oil-based diet (SO) during a 60-d pre-experimental period and subsequently fed either a ALA-rich linseed oil-based diet (LO) or an EPA- and DHA-rich fish oil diet (FO) in a 36-d experimental period. Fish growth and bioconversion capacity were evaluated at the end of both periods and on the 10th day of the experimental period, in order to determine the potential impact of a *n*-3 PUFA fish depletion on the apparent *in vivo* elongation and desaturation activities in fish fed on ALA. Three additional control groups included fish fed on SO (*n*-3 PUFA deficient diet), LO (ALA-rich diet) or FO (EPA- and DHA-rich diet) throughout the feeding trial.

## Methods

### Ethics statement

The experimental design of the feeding and digestibility trials was approved by the Animal Care and Use Committee of the Université catholique de Louvain (permit number 103203) as per the EU legal frameworks relating to the protection of animals used for scientific purposes (Directive 86/609/CEE) and guidelines of Belgian legislation governing the ethical treatment of animals (Decree M.B. 05.01.1994, 14 November 1993). Both *in vivo* experiments were conducted at the ‘Plateforme technologique et didactique en biologie aquicole Marcel Huet’ (Université catholique de Louvain), which is certified for animal services under the permit number LA 1220034. All manipulations were performed under anaesthesia and, if necessary, fish were euthanised using 2-phenoxyethanol at the required concentrations. All efforts were made to minimise fish numbers and suffering. No clinical symptoms were observed within or outside the experimental periods.

### Experimental diets

Experimental diets were formulated to differ in their fatty acid composition and contained either a high amount of 18 : 1*n*-9 for SO (blend of sunflower oils rich and low in 18 : 1*n*-9, 87:13, v/v), ALA for LO or *n*-3 LC-PUFA for FO. All diets were formulated to cover the fish requirement in LA, while avoiding any excess in that fatty acid, which might compete with ALA regarding desaturations and elongations. In practice, 18 : 1*n*-9-poor sunflower oil was included to all experimental diets (5 g/kg DM). A higher inclusion of 18 : 1*n*-9-poor sunflower oil was used for SO (15 g/kg DM as compared with 5 g/kg DM) in order to obtain a similar LA content in SO and LO. In addition, a sunflower oil rich in 18 : 1*n*-9 and poor in LA was added to SO at a 65 g/kg DM concentration to obtain a similar oil inclusion between all experimental diets. The experimental diets were formulated to meet the protein, vitamin and mineral requirements of rainbow trout^(^ ^5^ ^,^ ^35^ ^)^ (Table 1). The SO, LO and FO had a crude fat content of 94·1, 90·4 and 94·9 mg/g DM, respectively. This lipid content level was chosen in order to obtain a quick and efficient depletion in *n*-3 PUFA in the fish submitted to the SO treatment. Moreover, diets were formulated to obtain a targeted crude protein content of 520 mg/g DM and a targeted energy content of 20 MJ/kg DM. SO was deficient in *n*-3 PUFA (1·2 % of identified fatty acids), whereas LO was particularly rich in ALA (39·3 % of identified fatty acids, 99·8 % of *n*-3 PUFA), and FO rich in EPA and DHA (7·6 and 9 % of identified fatty acids, 33·7 and 40 % of *n*-3 PUFA, respectively) (Table 2). Chromic oxide was added at 10 g/kg DM to each experimental diets intended for the digestibility trial in order to serve as indigestible marker. The dry dietary components were mixed, homogenised using an electronic mixer (Kenwood Ltd), and extruded (HI 2251; Simplex). The diets were subsequently freeze-dried, manually crushed and then sieved to obtain pellets from 0·8 to 1·6 mm. The dry pellets were finally coated with oils and the diets were shaken several times for 48 h at 4°C before storage at −20°C until feeding or analysis.

**Table 1:** Components (g/kg DM) of the experimental diets formulated with sunflower oil, linseed oil or fish oil

	SO	LO	FO
Casein	288·3	288·3	288·3
Gelatin	50	50	50
Wheat gluten meal	250	250	250
Modified starch	161·7	161·7	161·7
Glucose	25	25	25
Agar	10	10	10
Carboxymethylcellulose	40	40	40
Cellulose	20	20	20
Vitamin premix	10	10	10
Mineral premix	65	65	65
18 : 1*n*-9-rich sunflower oil	65	0	0
18 : 1*n*-9-poor sunflower oil	15	5	5
Linseed oil	0	75	0
Cod liver oil	0	0	75

SO, sunflower oil-based diet; LO, linseed oil-based diet; FO, fish oil-based diet.

* Sigma-Aldrich.

† Vitamin complex (g/kg premix) according to Rollin *et al.* ^(^^35^ ^)^: retinol acetate 0·67, ascorbic acid 120, cholecalciferol 0·1, *α*-tocopherol acetate 34·2, menadione 2·2, thiamin 5·6, riboflavin 12, pyridoxine 4·5, calcium pantothenate 14·1, *p*-aminobenzoic acid 40, cyanocobalamin 0·03, niacin 30, biotin 0·1, choline chloride 350, folic acid 1·5, inositol 50, canthaxanthin 10, butylated hydroxytoluene 1·5, butylated hydroxyanisole 1·5, *α*-cellulose 322·1.

‡ Mineral complex (g/kg premix) according to Rollin *et al.* ^(^ ^35^ ^)^: CaHPO_4_.2H_2_O 295·5, Ca(H_2_PO_4_)_2_.H_2_O 217, NaHCO_3_ 94·5, Na_2_SeO_3_.5H_2_O 0·011, KCl 100, NaCl 172·4, KI 0·2, MgCl_2_ 63·7, MgSO_4_.7H_2_O 70·32, MnSO_4_.H_2_O 1·52, FeSO_4_.7H_2_O 12·41, CuSO_4_.5H_2_O 0·4, ZnSO_4_.7H_2_O 10.

§ Vandemoortele.

|| Lambert Chemicals.

¶ Certa.

**Table 2:** Fatty acid composition (mg/g DM) of the experimental diets

Fatty acids	SO	LO	FO
14 : 0	0·1	0·1	2·7
16 : 0	4·9	5·7	9·0
18 : 0	2·0	2·4	1·4
16 : 1*n*-7	0·1	0·1	3·4
18 : 1*n*-7	1·0	0·7	1·9
18 : 1*n*-9	48·4	13·2	11·6
20 : 1*n*-9	0·2	0·1	4·4
18 : 2*n*-6	15·9	17·6	9·7
18 : 3*n*-6	/	0·1	0·04
20 : 2*n*-6	/	0·02	0·2
20 : 3*n*-6	/	0·02	0·05
20 : 4*n*-6	/	/	0·2
22 : 4*n*-6	/	/	/
22 : 5*n*-6	/	/	0·1
18 : 3*n*-3	0·9	26·3	1·2
18 : 4*n*-3	/	/	0·9
20 : 3*n*-3	/	0·1	0·1
20 : 4*n*-3	/	/	0·5
20 : 5*n*-3	/	/	4·5
22 : 5*n*-3	/	/	0·8
22 : 6*n*-3	/	/	5·3
Total	74·4	66·9	59·1
ΣSFA*	7·7	8·5	13·5
ΣMUFA†	49·9	14·3	21·9
ΣC18 *n*-6 PUFA‡	15·9	17·8	9·7
Σ*n*-6 LC-PUFA§	0	0·04	0·6
ΣC18 *n*-3 PUFA||	0·9	26·3	2·1
Σ*n*-3 LC-PUFA¶	0	0·1	11·2
*n*-3:*n*-6**	0·1	1·5	1·3
*n*-3:*n*-6 LC-PUFA††	0	1·2	18·9

SO, sunflower oil-based diet; LO, linseed oil-based diet; FO, fish oil-based diet; LC-PUFA, long-chain PUFA.

* Sum of SFA, includes 20 : 0, 22 : 0 and 24 : 0.

† Sum of MUFA, includes 14 : 1*n*-5, 22 : 1*n*-9 and 24 : 1*n*-9.

‡ Sum of *n*-6 PUFA with 18C.

§ Sum of *n*-6 LC-PUFA with 20C and 22C.

|| Sum of *n*-3 PUFA with 18C.

¶ Sum of *n*-3 LC-PUFA with 20C and 22C.

** Ratio of total *n*-3 PUFA:total *n*-6 PUFA.

†† Ratio of *n*-3 LC-PUFA:*n*-6 LC-PUFA.

### Fish husbandry

Fertilised eggs from domesticated rainbow trout breeders were supplied by a commercial fish farm (La Fontaine aux Truites). After hatching, rainbow trout fry were fed a commercial diet for 2 months before the feeding trial. After 48 h of feed deprivation, rainbow trout fry (mean initial body weight 0·70 (sem 0·01) g/fish) were randomly distributed among seventeen tanks (11-litre capacity) to obtain 225 fish/tank. Fish of two tanks were sampled as an initial sample, weighed and stored at −20°C for subsequent analyses. Throughout the feeding trial, feeding was carried out by hand twice daily (08.30 and 16.00 hours) to apparent satiation (pellets from 0·8 to 1·6 mm, depending on the fish size). Fish were subjected to a 12 h light–12 h dark cycle photoperiod at a mean water temperature of 14°C with a 1 litre/min flow. From the 1st to the 60th feeding day, fish of nine tanks were fed on SO (*n* 9), three tanks were fed on LO (*n* 3) and three tanks were fed on FO (*n* 3). On the 20th day, fish were transferred to larger tanks (50 litre capacity) supplied by water at 11·5±0·5°C on a 5 litres/min flow basis. At the end of the 60-d pre-experimental period, considered to be long enough to highly reduce the *n*-3 PUFA content of fish fed on SO, six tanks previously held on SO were switched, either to LO (three tanks), or FO (three tanks). The second feeding period lasted 36 d. The experimental conditions were therefore named as SO, LO and FO for fish fed on SO, LO and FO (*n* 3), respectively, during 96 d, and as SO/LO and SO/FO (*n* 3) for fish fed on SO during the first 60-d pre-experimental period and then on LO or FO, respectively, during the second 36-d experimental period. Throughout the feeding trial, the biomass was determined every 10th feeding day after 48 h of feed deprivation. On days 60, 70 and 96, fish were weighed after 48 h of feed deprivation and fifteen fish of each tank were then euthanised with 2-phenoxyethanol, freeze-dried, homogenised and kept frozen (−20°C) until chemical analysis. At the end of the experimental period, the remaining fish from each tank fed their specific diet until the digestibility trial. The digestibility trial was performed with 5 (sem 0·05) kg of fish in circular tanks (130 litre capacity). Fish remained under experiment until accumulating sufficient faeces. The water was supplied at a 4 litres/min flow, the temperature was maintained at 11±1°C throughout the trial and fish were subjected to a 12 h light–12 h dark cycle photoperiod. Fish were fed manually twice daily (09.00 and 17.00 hours) to apparent satiation whilst avoiding any undesirable mixing of feed and faeces. Faeces were collected continuously through a rotating automatic faeces collector system^(^ ^36^ ^)^. Faeces collected per tank were weighed, freeze-dried, homogenised and stored at −20°C until further analyses.

### Chemical analysis

The DM and crude fat contents were analysed following analytical methods from the Association of Official Analytical Chemists^(^ ^37^ ^)^. In brief, DM was measured by drying samples at 105°C for 16 h and the crude fat content was evaluated using diethyl ether extraction according to Soxhlet method. The chromic oxide concentration in diets and faeces was determined following a protocol involving acid digestion followed by oxidation before photometric measurement (Cecil Instruments) at 350 nm^(^ ^38^ ^)^. The fatty acid composition of diets, fish and faeces was evaluated after lipid extraction of samples following the Folch method^(^ ^39^ ^)^ with subsequent modifications^(^ ^40^ ^)^. In brief, lipids from 1 g of dried sample were extracted by 60 ml of chloroform–methanol (2:1, v/v) (VWR Chemicals). Tridecanoic acid (Sigma-Aldrich) was used as internal standard for fatty acid quantification. The extracted fatty acids were converted into fatty acid methyl esters via methylation under alkaline conditions (KOH in methanol, 0·1 m, at 70°C for 60 min) and then under acidic conditions (HCl in methanol, 1·2 m, at 70°C for 20 min). The resultant fatty acid methyl esters were subsequently separated by GC. The GC Trace (Thermo Scientific) was equipped with an RT2560 capillary column (100 m×0·25 mm internal diameter, 0·2 µm film thickness; Restek), an ‘on column’ automatic injector and a flame ionisation detector kept at a constant temperature of 255°C. The system used H as the carrier gas at an operating pressure of 200 kPa. The oven temperature programme was as follows: an initial temperature of 80°C, which progressively increased at 25°C/min up to 175°C, a holding temperature of 175°C during 25 min followed by an increase at 10°C/min up to 205°C, a holding temperature of 205°C during 4 min followed by an increase at 10°C/min up to 225°C and a holding temperature of 225°C during 20 min. Each peak was identified by comparison of retention times with those for pure methyl ester standards (Larodan and Nu-Check Prep). Data processing was operated via ChromQuest software 3.0 (Thermo Finnigan). The final results are expressed in mg/g DM.

### Performance parameters and fatty acid metabolism computation

Daily growth coefficient (DGC) was calculated as follows: DGC (g^1/3^/d×1000)=1000×((final fish weight (g))^1/3^−(initial fish weight (g))^1/3^)/feeding d. Daily feed intake was calculated as the percentage of biomass. Feed efficiency (FE, g/g DM) was calculated as the ratio between fish weight gain (g) and dry feed intake (g DM). The apparent fatty acid digestibility was assessed using the standard formula: 100−(100×(Cr_2_O_3_ in diet (mg/g DM))/(Cr_2_O_3_ in faeces (mg/g DM))×(fatty acid in faeces (mg/g DM))/(fatty acid in diet (mg/g DM))). The coefficient of distance (CD) was implemented to compare fatty acid concentrations between two treatments and was calculated as previously described^(^ ^41^ ^)^. The estimation of the apparent *in vivo* fatty acid metabolism was calculated via the implementation of the whole body fatty acid balance method, as initially proposed and described by Turchini *et al*.^(^ ^42^ ^)^ and later modified^(^ ^20^ ^,^ ^43^ ^)^.

### Statistical analysis

All data are presented as mean values with their standard errors (*n* 2, 3 or 9, as stated). Before statistical analysis, data were subjected to log or square root transformation if identified as non-homogenous (Levene’s test) to meet the assumptions for statistical methods. The significance of difference between dietary treatments was determined using one-way ANOVA at a significance level of *α* 5 %, followed by Tukey’s (parametric with *α* 5 %) or Wilcoxon’s (non-parametric with *α* 1·69 %) *post hoc* tests. Statistical analysis was carried out using JMP^®^ Pro 12 (SAS).

## Results

### Fish growth performance

**Fig. 1:**
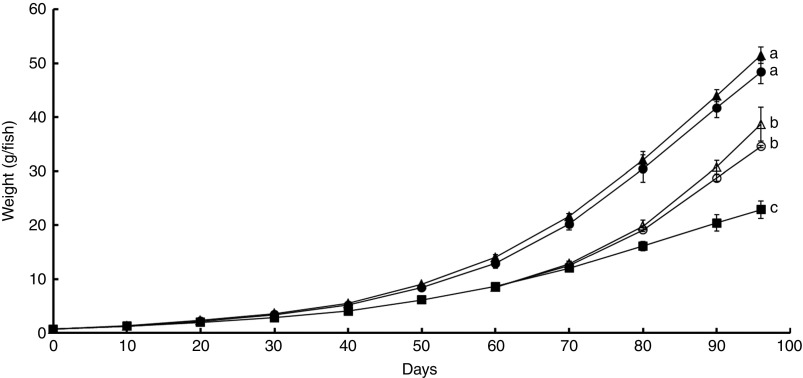
Mean weight (g/fish) of rainbow trout at each sampling time point over the 60-d pre-experimental period and the 36-d experimental period. Values are means (*n* 3 except sunflower oil-based diet (SO) treatment from starting day until day 60 for which *n* 9), with their standard errors. The fish weight was impacted by the dietary treatment regarding the lowest weight of fish fed SO (■) and the highest weights of fish fed on linseed oil–based diet (LO, ●) or fish oil-based diet (FO, ▲) during 96 d. Intermediate fish weights were reported when feeding fish on SO for 60 d and then on LO (SO/LO, ○) or FO (SO/FO, Δ) for 36 d. ^a,b,c^ Mean values with unlike superscript letters were significantly different (Tukey’s *post hoc* test, *α* 5 %).

The experimental diets were readily accepted by fish and mortality throughout the feeding trial was low and unrelated to the dietary treatment (mean mortality rate <0·01 %/d). In contrast, fish weight and growth performance were highly impacted by the dietary lipid source. Fish fed on SO throughout the feeding trial recorded the lowest final weight (22·9 (sem 0·9) g/fish) whereas fish fed on LO and FO recorded the highest final weights (48·4 (sem 1·2) and 51·5 (sem 0·9) g/fish, respectively) (Fig. 1). This trend manifested further in decreased DGC and FE in fish subjected to the SO treatment over the course of the feeding trial (Table 3). In LO fish, a reduced DGC was noticed in comparison to fish fed on FO at the end of the 60-d pre-experimental period, but not at the end of the feeding trial. The replacement of SO by LO or FO for 36 d also induced significant differences. The SO/LO and SO/FO final fish weights were higher than those of fish fed on SO for 96 d, but did not reach those of fish constantly fed on LO and FO for 96 d (Fig. 1). DGC values were also higher for the SO/LO and SO/FO treatments as compared with the SO treatment, and similar to those observed for the LO and FO fish groups (Table 3). Moreover, an increased FE was recorded for SO/LO and SO/FO fish as compared with SO fish. These increased FE were similar to those of fish fed on LO and FO for 96 d. The SO/LO fish had a significantly reduced DGC as compared with the SO/FO fish group but similar final fish weights, feed intake and FE.

**Table 3:** Growth performance of rainbow trout fed on diets differing in fatty acid composition during a 60-d pre-experimental feeding period (days 1–60) followed by a 36-d experimental period (days 61–96) (Mean values with their standard errors; *n* 3 except for sunflower oil-based diet (SO) for days 1 to 60 period (*n* 9))

	SO		LO		FO		SO/LO		SO/FO	
	Mean	sem	Mean	sem	Mean	sem	Mean	sem	Mean	sem
Days 1–60										
DGC (g^1/3^/d × 1000)	19·3^c^	0·1	24·2^b^	0·5	25·4^a^	0·3	/	/	/	/
Feed intake (%/d)	2·9^b^	0·0	3·2^a^	0·1	3·1^a^	0·0	/	/	/	/
FE	1·4^b^	0·0	1·5^a,b^	0·1	1·6^a^	0·0	/	/	/	/
Days 1–96										
DGC (g^1/3^/d × 1000)	20·3^b^	0·4	28·7^a^	0·3	29·5^a^	0·2	/	/	/	/
Feed intake (%/d)	2·8^b^	0·0	3·0^a^	0·1	2·9^a,b^	0·0	/	/	/	/
FE	1·1^b^	0·0	1·3^a^	0·0	1·4^a^	0·0	/	/	/	/
Days 61–96										
DGC (g^1/3^/d × 1000)	21·9^c^	0·7	36·1^a,b^	0·0	36·4^a,b^	0·9	33·7^b^	0·1	37·3^a^	1·2
Feed intake (%/d)	2·5^c^	0·0	2·7^b,c^	0·0	2·5^c^	0·1	2·8^a,b^	0·0	2·9^a^	0·0
FE	1·0^b^	0·0	1·3^a^	0·0	1·4^a^	0·0	1·3^a^	0·0	1·4^a^	0·0

LO, linseed oil-based diet; FO, fish oil-based diet; SO/LO, SO until day 60 and then LO from days 61–96; SO/FO, SO until day 60 and then FO from days 61–96; DGC, daily growth coefficient; FE, feed efficiency. ^a,b,c^ Mean values within a row with unlike superscript letters were significantly different (Tukey’s (parametric, *α* 5 %) or Wilcoxon’s (non-parametric, *α* 1·69 %) *post hoc* tests).

### Fish fatty acid composition

Throughout the feeding trial, fish fed on LO recorded the highest C18 *n*-6 PUFA concentration while fish fed on SO recorded the highest *n*-6 LC-PUFA concentration (Tables 4 and 5), despite the absence of *n*-6 LC-PUFA in SO (Table 2). The pre-experimental period induced a high depletion in *n*-3 PUFA for fish fed on SO as these recorded the lowest concentrations of C18 *n*-3 PUFA and *n*-3 LC-PUFA (0·79 (sem 0·10) and 2·75 (sem 0·47) mg/g DM throughout the feeding trial, respectively). In contrast, the highest C18 *n*-3 PUFA and *n*-3 LC-PUFA concentrations were, respectively, reported in fish fed on LO (42·68 (sem 0·74) mg/g DM) and in fish fed on FO (25·82 (sem 0·94) mg/g DM) (Tables 4 and 5). Concentrations of 18 : 4*n*-3, 20 : 3*n*-3 and 20 : 4*n*-3 were significantly higher in fish fed on LO, while fish fed on FO recorded the highest EPA and DHA concentrations. On the 10th day of the experimental period (day 70), the SO/LO fish recovered 57 % (CD 3·9) of the *n*-3 LC-PUFA found in fish fed on LO for 70 d. Similarly, the SO/FO fish recovered 51 % (CD 9) of the *n*-3 LC-PUFA found in fish fed on FO for 70 d (Table 5). At the end of the experimental period, the *n*-3 LC-PUFA of SO/LO and SO/FO fish almost reached those of fish fed on LO and FO with a recovery rate of 82 % (CD 1·8) and 84 % (CD 2·9), respectively. In terms of DHA, the SO/LO fish recovered 62 % (CD 3·4) and 84 % (CD 1·6) of the DHA found in fish fed on LO on the 10th day and at the end of the 36-d experimental period, respectively. The SO/FO fish recovered 49 % (CD 8·6) and 85 % (CD 2·8) of the DHA found in fish fed on FO, on the 10th day and at the end of the experimental period, respectively.

**Table 4:** Fatty acid composition (mg/g DM) of fish held on dietary treatments differing in the dietary lipid source on the starting and at the end of the 60-d pre-experimental feeding period (Mean values with their standard errors; *n* 3 except initial treatment (*n* 2))

			Day 60					
	Initial		SO		LO		FO	
Fatty acids	Mean	sem	Mean	sem	Mean	sem	Mean	sem
14 : 0	7·65	0·18	2·83^b^	0·01	2·30^c^	0·09	7·08^a^	0·16
16 : 0	26·75	0·67	30·79^c^	0·08	33·12^b^	0·36	39·01^a^	1·21
18 : 0	4·74	0·39	8·31^b^	0·07	10·26^a^	0·26	8·05^b^	0·36
16 : 1*n*-7	8·09	0·17	10·00^b^	0·09	9·72^b^	0·27	15·90^a^	0·56
18 : 1*n*-7	4·08	0·04	4·28^b^	0·10	3·79^b^	0·10	6·82^a^	0·22
18 : 1*n*-9	17·51	0·53	124·74^a^	0·89	59·00^b^	0·92	49·44^c^	1·20
20 : 1*n*-9	8·70	0·18	4·36^b^	0·07	2·00^c^	0·02	9·78^a^	0·37
18 : 2*n*-6	5·08	0·26	25·37^b^	0·33	36·33^a^	0·81	22·91^b^	0·74
18 : 3*n*-6	0·13	0·00	2·77^a^	0·05	1·23^b^	0·09	0·43^c^	0·01
20 : 2*n*-6	0·48	0·00	1·25^b^	0·03	1·58^a^	0·05	1·54^a^	0·07
20 : 3*n*-6	0·25	0·01	2·32^a^	0·02	1·69^b^	0·07	1·04^c^	0·01
20 : 4*n*-6	1·30	0·02	3·38^a^	0·02	0·97^b^	0·03	1·03^b^	0·01
22 : 4*n*-6	0·05	0·01	0·41^a^	0·02	0·06^b^	0·00	0·08^b^	0·01
22 : 5*n*-6	0·29	0·05	3·24^a^	0·07	0·20^c^	0·01	0·32^b^	0·01
18 : 3*n*-3	2·47	0·20	0·64^c^	0·05	36·27^a^	0·74	2·15^b^	0·06
18 : 4*n*-3	2·72	0·13	0·35^c^	0·01	4·94^a^	0·33	1·23^b^	0·02
20 : 3*n*-3	0·62	0·04	0·00^c^	0·00	1·66^a^	0·10	0·40^b^	0·03
20 : 4*n*-3	1·53	0·07	0·06^c^	0·00	2·11^a^	0·09	1·00^b^	0·06
20 : 5*n*-3	10·62	0·25	0·58^c^	0·02	2·31^b^	0·09	4·61^a^	0·11
22 : 5*n*-3	2·55	0·05	0·15^c^	0·01	0·79^b^	0·02	1·49^a^	0·05
22 : 6*n*-3	30·57	0·76	2·67^c^	0·00	10·33^b^	0·23	17·77^a^	0·62
Total	139·94	4·05	230·97^a^	1·44	222·07^a^	3·55	195·42^b^	5·82
ΣSFA*	39·54	1·24	43·37	0·10	46·32	0·21	54·67	1·75
ΣMUFA†	41·74	0·96	144·40^a^	1·05	75·28^c^	1·22	84·76^b^	2·44
ΣC18 *n*-6 PUFA‡	5·21	0·26	28·14^b^	0·29	37·56^a^	0·88	23·33^c^	0·74
Σ*n*-6 LC-PUFA§	2·36	0·09	10·60^a^	0·10	4·50^b^	0·09	4·00^c^	0·10
ΣC18 *n*-3 PUFA||	5·19	0·34	0·99^c^	0·05	41·21^a^	0·92	3·38^b^	0·08
Σ*n*-3 LC-PUFA¶	45·89	1·17	3·47^c^	0·03	17·21^b^	0·47	25·27^a^	0·79
*n*-3:*n*-6**	6·75	0·11	0·12^c^	0·00	1·39^a^	0·00	1·05^b^	0·00
*n*-3:*n*-6 LC-PUFA††	19·44	0·22	0·33^c^	0·00	3·83^b^	0·04	6·31^a^	0·04

SO, sunflower oil-based diet; LO, linseed oil-based diet; FO, fish oil-based diet; LC-PUFA, long-chain PUFA (≥20C). ^a,b,c^ Mean values within a row with unlike superscript letters were significantly different (Tukey’s (parametric, *α* 5 %) or Wilcoxon’s (non-parametric, *α* 1·69 %) *post hoc* tests on square root transformed final condition values).

* Sum of SFA, includes 20 : 0, 22 : 0 and 24 : 0.

† Sum of MUFA, includes 14 : 1*n*-5, 22 : 1*n*-9 and 24 : 1*n*-9.

‡ Sum of *n*-6 PUFA with 18C.

§ Sum of *n*-6 LC-PUFA with 20C and 22C.

|| Sum of *n*-3 PUFA with 18C.

¶ Sum of *n*-3 LC-PUFA with 20C and 22C.

** Ratio of total *n*-3 PUFA:total *n*-6 PUFA.

†† Ratio of *n*-3 LC-PUFA:*n*-6 LC-PUFA.

**Table 5:** Fatty acid composition (mg/g DM) of fish held on dietary treatments differing in dietary lipid source on the 10th (day 70) and the end (day 96) of the 36-d experimental period (Mean values with their standard errors; *n* 3 except sunflower oil-based diet (SO) until day 60 and then fish oil-based diet (FO) from days 61–96 (SO/FO) treatment at day 70 (*n* 2))

	Day 70										Day 96									
	SO		LO		FO		SO/LO		SO/FO		SO		LO		FO		SO/LO		SO/FO	
Fatty acids	Mean	sem	Mean	sem	Mean	sem	Mean	sem	Mean	sem	Mean	sem	Mean	sem	Mean	sem	Mean	sem	Mean	sem
14 : 0	2·96^c^	0·08	2·32^d^	0·05	7·19^a^	0·08	2·77^c^	0·09	4·45^b^	0·00	2·53^r^	0·09	2·74^r^	0·06	7·53^p^	0·03	2·53^r^	0·06	6·52^q^	0·13
16 : 0	30·22^b^	0·90	32·71^b^	1·38	40·46^a^	0·54	32·04^b^	1·31	33·13^b^	0·26	28·53^s^	0·53	39·06^q^	0·69	45·01^p^	0·25	35·19^r^	0·54	41·31^q^	0·64
18 : 0	8·65^b^	0·26	10·78^a^	0·53	8·96^b^	0·16	9·68^a,b^	0·38	8·05^b^	0·01	8·99^s^	0·13	12·98^p^	0·24	9·96^r^	0·09	11·52^q^	0·17	9·26^s^	0·08
16 : 1*n*-7	9·60^b,c^	0·43	9·37^c^	0·49	16·20^a^	0·26	9·39^b,c^	0·48	11·58^b^	0·08	8·31^r^	0·18	11·54^q^	0·43	17·92^p^	0·32	10·33^q^	0·47	15·91^p^	0·43
18 : 1*n*-7	3·64^c^	0·16	3·34^c^	0·22	6·51^a^	0·09	3·61^c^	0·10	4·66^b^	0·02	3·57^s^	0·13	4·04^r^	0·11	7·52^p^	0·09	3·92^r,s^	0·06	6·67^q^	0·08
18 : 1*n*-9	120·29^a^	4·53	58·29^c^	2·85	50·86^c^	0·80	98·20^b^	4·17	87·13^b^	0·50	118·16^p^	1·61	69·65^r^	1·22	57·23^s^	0·62	75·03^q^	0·17	65·71^r^	0·66
20 : 1*n*-9	4·36^c^	0·14	2·11^e^	0·16	9·66^a^	0·17	3·57^d^	0·08	6·14^b^	0·03	4·60^r^	0·12	2·41^s^	0·09	10·88^p^	0·19	2·55^s^	0·06	9·55^q^	0·21
18 : 2*n*-6	24·56^c^	0·81	33·88^a^	1·01	22·32^c^	0·31	29·14^a,b^	0·91	22·97^c^	0·13	24·43^r^	0·38	38·36^p^	0·42	24·68^r^	0·34	35·84^q^	0·39	24·75^r^	0·45
18 : 3*n*-6	2·68^a^	0·16	1·27^c^	0·05	0·62^d^	0·01	2·18^b^	0·06	1·98^b^	0·07	2·39^p^	0·02	1·22^r^	0·01	0·47^t^	0·02	1·50^q^	0·04	0·85^s^	0·01
20 : 2*n*-6	1·32^c^	0·04	1·71^a^	0·09	1·58^a,b^	0·00	1·49^a,b,c^	0·03	1·42^b,c^	0·05	1·39^s^	0·03	2·04^p^	0·01	1·83^q,r^	0·02	1·88^q^	0·02	1·74^r^	0·03
20 : 3*n*-6	2·15^a^	0·17	1·66^b^	0·10	0·98^c^	0·02	2·07^a,b^	0·06	1·67^a,b^	0·03	2·15^p^	0·04	1·83^q^	0·05	1·02^s^	0·02	1·85^q^	0·02	1·25^r^	0·01
20 : 4*n*-6	3·29^a^	0·10	0·96^c^	0·06	0·97^c^	0·02	2·57^b^	0·10	2·46^b^	0·07	3·42^p^	0·03	1·16^r^	0·02	1·13^r^	0·02	1·58^q^	0·01	1·51^q^	0·02
22 : 4*n*-6	0·46^a^	0·01	0·09^c^	0·01	0·08^c^	0·00	0·33^b^	0·01	0·30^b^	0·01	0·46^p^	0·02	0·09^r^	0·01	0·10^r^	0·00	0·15^q^	0·01	0·16^q^	0·01
22 : 5*n*-6	3·08^a^	0·07	0·65^c^	0·08	0·69^c^	0·05	2·22^b^	0·08	2·37^b^	0·14	3·60^p^	0·08	0·20^s^	0·01	0·33^r^	0·01	0·70^q^	0·04	0·83^q^	0·03
18 : 3*n*-3	0·45^d^	0·07	38·37^a^	0·89	1·91^c^	0·03	15·94^b^	0·32	1·44^c^	0·17	0·51^s^	0·09	38·75^p^	1·04	2·05^r^	0·06	31·26^q^	1·07	1·85^r^	0·09
18 : 4*n*-3	0·22^e^	0·01	4·94^a^	0·05	1·25^c^	0·01	1·99^b^	0·07	0·70^d^	0·03	0·20^s^	0·02	4·76^p^	0·04	1·34^r^	0·04	3·89^q^	0·17	1·13^r^	0·03
20 : 3*n*-3	0·01^e^	0·00	1·84^a^	0·07	0·26^c^	0·01	0·76^b^	0·01	0·13^d^	0·00	0·01^r^	0·01	2·12^p^	0·08	0·33^q^	0·02	1·72^p^	0·08	0·28^q^	0·04
20 : 4*n*-3	0·37^c^	0·11	2·27^a^	0·24	1·35^b^	0·04	1·15^b^	0·05	1·15^b^	0·22	0·08^t^	0·01	2·30^p^	0·07	1·36^r^	0·06	1·77^q^	0·01	1·04^s^	0·03
20 : 5*n*-3	0·44^d^	0·01	2·07^b^	0·11	4·48^a^	0·09	1·17^c^	0·03	2·13^b^	0·02	0·26^t^	0·01	2·39^r^	0·03	5·22^p^	0·06	1·91^s^	0·03	4·40^q^	0·11
22 : 5*n*-3	0·11^d^	0·01	0·86^b^	0·09	1·47^a^	0·04	0·44^c^	0·02	0·70^b^	0·01	0·05^t^	0·01	0·97^r^	0·03	1·72^p^	0·03	0·73^s^	0·02	1·43^q^	0·05
22 : 6*n*-3	1·99^d^	0·05	9·01^b^	0·59	16·96^a^	0·45	5·57^c^	0·24	8·32^b^	0·01	1·44^t^	0·07	9·97^r^	0·17	19·01^p^	0·38	8·42^s^	0·17	16·22^q^	0·53
Total	223·09	7·91	219·76	9·00	197·81	2·98	228·19	8·40	205·28	0·01	217·47^r^	2·93	250·05^p^	3·28	220·29^r^	1·94	236·01^q^	2·51	215·80^r^	2·51
ΣSFA*	43·01^b^	1·28	46·32^b^	1·99	57·12^a^	0·77	45·44^b^	1·85	46·50^b^	0·27	41·43^s^	0·76	55·42^q^	0·89	63·11^p^	0·37	50·09^r^	0·51	57·78^q^	0·83
ΣMUFA†	138·93^a^	5·26	73·86^c^	3·79	85·76^c^	1·30	115·72^b^	4·88	111·02^b^	0·65	135·63^p^	1·99	88·48^s^	1·51	96·58^q,r^	1·11	92·72^r,s^	0·42	100·59^q^	1·00
ΣC18 *n*-6 PUFA‡	27·25^b^	0·97	35·16^a^	1·05	22·94^c^	0·32	31·32^a^	0·95	24·95^b,c^	0·06	26·81^r^	0·36	39·58^p^	0·43	25·15^r^	0·33	37·35^q^	0·42	25·60^r^	0·46
Σ*n*-6 LC-PUFA§	10·29^a^	0·40	5·07^c^	0·32	4·30^c^	0·06	8·68^b^	0·25	8·22^b^	0·02	11·02^p^	0·11	5·31^r^	0·03	4·41^s^	0·03	6·16^q^	0·07	5·49^r^	0·06
ΣC18 *n*-3 PUFA||	0·68^e^	0·08	43·32^a^	0·94	3·17^c^	0·03	17·94^b^	0·36	2·14^d^	0·20	0·71^s^	0·10	43·51^p^	1·03	3·39^r^	0·06	35·15^q^	1·24	2·98^r^	0·07
Σ*n*-3 LC-PUFA¶	2·93^e^	0·18	16·04^b^	1·08	24·53^a^	0·62	9·10^d^	0·25	12·44^c^	0·26	1·86^t^	0·10	17·75^r^	0·17	27·65^p^	0·52	14·54^s^	0·11	23·36^q^	0·64
*n*-3:*n*-6**	0·10^e^	0·00	1·48^a^	0·01	1·02^b^	0·01	0·68^c^	0·01	0·44^d^	0·02	0·07^t^	0·01	1·36^p^	0·01	1·05^r^	0·01	1·14^q^	0·02	0·85^s^	0·00
*n*-3:*n*-6 LC-PUFA††	0·28^e^	0·02	3·16^b^	0·06	5·71^a^	0·11	1·05^d^	0·01	1·51^c^	0·04	0·17^t^	0·01	3·34^r^	0·02	6·27^p^	0·11	2·36^s^	0·01	4·25^q^	0·08

LO, linseed oil-based diet; SO/LO, SO until day 60 and then LO from days 61–96; LC-PUFA, long-chain PUFA (≥20C). ^a,b,c,d,e^ For day 70 and ^p,q,r,s,t^ for day 96: mean values within a row with unlike superscript letters were significantly different (Tukey’s *post hoc* test on square root transformed values for each sampling day, *α* 5 %).

* Sum of SFA, includes 20 : 0, 22 : 0 and 24 : 0.

† Sum of MUFA, includes 14 : 1*n*-5, 22 : 1*n*-9 and 24 : 1*n*-9.

‡ Sum of *n*-6 PUFA with 18C.

§ Sum of *n*-6 LC-PUFA with 20C and 22C.

|| Sum of *n*-3 PUFA with 18C.

¶ Sum of *n*-3 LC-PUFA with 20C and 22C.

** Ratio of total *n*-3 PUFA:total *n*-6 PUFA.

†† Ratio of *n*-3 LC-PUFA:*n*-6 LC-PUFA.

### *In vivo* fatty acid metabolism

Over the course of the entire feeding trial, total apparent *in vivo* SFA and MUFA elongation and Δ-9 desaturation activities were highest in fish subjected to the LO and FO treatments, while fish receiving SO recorded the highest total apparent *in vivo* SFA and MUFA *β*-oxidation (Tables 6 and 7). Within the *n*-6 PUFA family, fish fed on SO demonstrated a higher apparent *in vivo* elongation as well as higher Δ-5 and Δ-6 desaturation activities in comparison to those fed on LO and FO (Tables 6 and 7). In contrast, within the *n*-3 PUFA family, the highest apparent *in vivo* elongation, Δ-5 and Δ-6 desaturation activities were displayed in fish subjected to the LO treatment (Table 6). The apparent *in vivo* activities of fish at the end and on the 10th day of the experimental period are reported in Tables 7 and 8, respectively. At the end of the experimental period, fish of the SO/LO group recorded lower apparent *in vivo* enzyme activities as compared with fish fed on LO at each elongation and desaturation step of the *n*-6 pathway. Similar observations were reported on the 10th day of the experimental period, but only significantly for the apparent *in vivo* elongation activity (Table 8). In contrast, no differences in apparent *in vivo* elongation and desaturation activities within the *n*-3 pathway were observed between SO/LO and LO treatments, at the end of the trial or on the 10th day of the experimental period (Tables 7 and 8). Considering both *n*-6 and *n*-3 pathways, similar apparent *in vivo* Δ-5 and Δ-6 desaturation activities were reported between SO/LO and LO fish groups. With respect to the dietary replacement of SO by FO (SO/FO), no statistical differences in apparent *in vivo n*-6 and *n*-3 PUFA enzyme activities were seen between SO/FO and FO fish groups on the 10th day and at the end of the experimental period (Tables 7 and 8).

**Table 6:** Fatty acid metabolism (nmol/g per d), deduced by the whole body fatty acid balance method, of rainbow trout held on varying dietary lipid source diets for a 60-d pre-experimental feeding period (Mean values with their standard errors; *n* 3)

	SO		LO		FO	
	Mean	sem	Mean	sem	Mean	sem
SFA and MUFA						
*β*-Oxidation	223·5^a^	26·4	4·7^b^	0·8	26·0^b^	10·1
Elongation	1865·3^b^	10·4	3850·3^a^	191·0	3205·0^a^	225·6
Δ-9 desaturation	286·6^c^	3·6	921·1^a^	48·4	722·3^b^	49·0
*n*-6 PUFA						
*β*-Oxidation	173·0^a^	8·1	179·2^a^	26·0	4·8^b^	4·8
Elongation	379·1^a^	5·4	111·6^b^	2·7	58·5^c^	2·6
****18 : 3*n*-6 to 20 : 3*n*-6	205·9^a^	1·7	66·0^b^	2·1	31·2^c^	0·7
****20 : 4*n*-6 to 22 : 4*n*-6	77·1^a^	1·6	5·4^b^	0·2	2·1^c^	0·4
****22 : 4*n*-6 to 24 : 4*n*-6	68·4^a^	1·4	4·2^b^	0·3	0·5^c^	0·2
Δ-5 desaturation	153·1^a^	1·3	27·4^b^	0·9	10·2^c^	0·5
Δ-6 desaturation	344·0^a^	2·0	92·2^b^	3·6	40·2^c^	0·9
****18 : 2*n*-6 to 18 : 3*n*-6	275·6^a^	1·0	88·0^b^	3·6	39·6^c^	0·7
****24 : 4*n*-6 to 24 : 5*n*-6	68·4^a^	1·4	4·2^b^	0·3	0·5^c^	0·2
*n*-3 PUFA						
*β*-Oxidation	38·4^c^	0·8	476·2^a^	41·1	198·9^b^	13·2
Elongation	37·8^c^	2·0	761·9^a^	19·3	108·0^b^	22·4
****18 : 4*n*-3 to 20 : 4*n*-3	10·1^b^	1·1	309·7^a^	7·8	0·0^c^	0·0
****20 : 5*n*-3 to 22 : 5*n*-3	13·7^c^	0·5	215·7^a^	4·8	43·6^b,c^	11·8
****22 : 5*n*-3 to 24 : 5*n*-3	14·0^c^	0·4	200·6^a^	4·6	60·6^b^	11·0
Δ-5 desaturation	11·0^b^	1·0	260·5^a^	5·8	0^c^	0
Δ-6 desaturation	28·4^c^	1·3	637·9^a^	16·7	60·6^b^	11·0
****18 : 3*n*-3 to 18 : 4*n*-3	14·4^b^	1·0	437·3^a^	13·2	0·0^c^	0·0
****24 : 5*n*-3 to 24 : 6*n*-3	14·0^c^	0·4	200·6^a^	4·6	60·6^b^	11·0
*n*-6 and *n*-3 PUFA						
**** Δ-5 desaturation	164·1^b^	2·2	287·9^a^	6·7	10·2^c^	0·5
**** Δ-6 desaturation	372·5^b^	3·3	730·1^a^	20·2	100·8^c^	11·9

SO, sunflower oil-based diet; LO, linseed oil-based diet; FO, fish oil-based diet. ^a,b,c^ Mean values within a row with unlike superscript letters were significantly different (Tukey’s *post hoc* test on square root transformed values, *α* 5 %).

**Table 7:** Fatty acid metabolism (nmol/g per d), deduced by the whole body fatty acid balance method, of rainbow trout held on varying dietary lipid source diets for a 36-d experimental period after a 60-d pre-experimental period (Mean values with their standard errors; *n* 3)

	SO		LO		FO		SO/LO		SO/FO	
	Mean	sem	Mean	sem	Mean	sem	Mean	sem	Mean	sem
SFA and MUFA										
*β*-Oxidation	1545·9^a^	128·6	4·6^c^	0·8	10·3^b^	1·4	5·1^c^	0·8	12·5^b^	0·4
Elongation	1006·4^c^	100·6	6824·6^a^	361·9	5580·6^a,b^	359·3	4889·1^b^	272·2	4520·6^b^	243·8
Δ-9 desaturation	160·0^c^	7·9	1582·2	85·3	1234·9^b^	88·9	1078·9^b^	67·2	944·9^b^	60·3
*n*-6 PUFA										
*β*-Oxidation	548·7^a^	26·4	159·3^b^	27·0	0·0^c^	0·0	212·1^b^	40·4	1·8^c^	0·3
Elongation	348·9^a^	7·7	162·3^b^	2·9	82·9^d^	3·8	134·4^c^	4·4	78·7^d^	1·2
****18 : 3*n*-6 to 20 : 3*n*-6	180·3^a^	3·4	91·3^b^	2·4	40·3^d^	1·7	76·7^c^	2·7	36·4^d^	1·0
****20 : 4*n*-6 to 22 : 4*n*-6	74·7^a^	2·7	7·6^b^	0·4	3·4^c^	0·3	2·3^c^	0·7	2·6^c^	0·3
****22 : 4*n*-6 to 24 : 4*n*-6	66·6^a^	2·2	5·0^b^	0·2	0·7^c^	0·2	0·3^c,d^	0·3	0·0^d^	0·0
Δ-5 desaturation	141·5^a^	3·0	41·3^b^	1·2	16·5^d^	0·4	31·2^c^	1·2	12·6^e^	0·2
Δ-6 desaturation	293·1^a^	5·9	120·6^b^	4·3	52·4^d^	2·5	98·8^c^	4·8	43·5^d^	1·4
****18 : 2*n*-6 to 18 : 3*n*-6	226·4^a^	3·7	115·6^b^	4·1	51·7^d^	2·2	98·5^c^	4·6	43·5^d^	1·4
****24 : 4*n*-6 to 24 : 5*n*-6	66·6^a^	2·2	5·0^b^	0·2	0·7^c^	0·2	0·3^c,d^	0·3	0·0^d^	0·0
*n*-3 PUFA										
*β*-Oxidation	47·2^c^	4·5	485·3^a^	59·3	142·4^b^	15·7	496·2^a^	64·6	157·7^b^	4·4
Elongation	40·5^c^	6·1	1002·7^a^	26·4	239·4^b^	25·4	1010·9^a^	19·1	258·1^b^	20·8
****18 : 4*n*-3 to 20 : 4*n*-3	15·1^b^	2·3	412·0^a^	9·4	3·1^c^	1·6	410·4^a^	7·2	0·0^c^	0·0
****20 : 5*n*-3 to 22 : 5*n*-3	12·4^c^	1·8	279·2^a^	7·9	110·9^b^	12·4	283·1^a^	6·0	119·9^b^	11·4
****22 : 5*n*-3 to 24 : 5*n*-3	12·6^c^	1·8	253·1^a^	7·5	123·0^b^	11·6	260·1^a^	6·5	134·6^b^	11·0
Δ-5 desaturation	13·4^b^	2·1	346·5^a^	9·9	0·0^c^	0·0	347·4^a^	6·4	0·0^c^	0·0
Δ-6 desaturation	30·2^c^	4·5	808·6^a^	19·3	123·0^b^	11·6	821·7^a^	17·2	134·6^b^	11·0
****18 : 3*n*-3 to 18 : 4*n*-3	17·6^b^	2·7	555·5^a^	12·1	0·0^c^	0·0	561·5^a^	12·2	0·0^c^	0·0
****24 : 5*n*-3 to 24 : 6*n*-3	12·6^c^	1·8	253·1^a^	7·5	123·0^b^	11·6	260·1^a^	6·5	134·6^b^	11·0
*n*-6 and *n*-3 PUFA										
Δ-5 desaturation	154·9^b^	3·0	387·8^a^	10·8	16·5^c^	0·4	378·6^a^	7·2	12·6^c^	0·2
Δ-6 desaturation	323·3^b^	5·7	929·2^a^	23·4	175·4^c^	12·3	920·5^a^	21·3	178·1^c^	10·3

SO, sunflower oil-based diet; LO, linseed oil-based diet; FO, fish oil-based diet; SO/LO, SO until day 60 and then LO from days 61–96; SO/FO, SO until day 60 and then FO from days 61–96. ^a,b,c,d,e^ Mean values within a row with unlike superscript letters were significantly different (Tukey’s *post hoc* test on square root transformed values, *α* 5 %).

**Table 8:** Fatty acid metabolism (nmol/g per d), deduced by the whole body fatty acid balance method, of rainbow trout held on varying dietary lipid source diets for a 10-d experimental period after a 60-d pre-experimental period (Mean values with their standard errors; *n* 3 except sunflower oil-based diet (SO) until day 60 and then fish oil-based diet (FO) from days 61–96 (SO/FO) treatment (*n* 2))

	SO		LO		FO		SO/LO		SO/FO	
	Mean	sem	Mean	sem	Mean	sem	Mean	sem	Mean	sem
SFA and MUFA										
*β*-Oxidation	1101·5	421·5	20·6	7·0	63·0	29·9	355·2	333·1	685·1	99·4
Elongation	1994·9	374·3	6025·3	1511·3	6072·1	355·9	3045·2	964·3	1702·3	220·8
Δ-9 desaturation	273·5^b^	53·9	1354·2^a^	340·3	1281·6^a^	61·8	506·5^a,b^	185·0	192·9^b^	9·8
*n*-6 PUFA										
*β*-Oxidation	463·6^a^	111·2	401·2^a^	118·7	12·9^b^	12·9	454·2^a^	135·7	200·3^a,b^	43·0
Elongation	372·1^a^	45·4	305·6^a,b^	42·0	180·0^b,c^	19·0	142·6^c^	23·4	107·3^c^	34·5
****18 : 3*n*-6 to 20 : 3*n*-6	201·4^a^	29·9	141·5^a,b^	21·3	70·4^b,c^	5·0	75·2^b,c^	16·4	28·0^c^	9·8
****20 : 4*n*-6 to 22 : 4*n*-6	73·2^a^	6·5	50·9^a,b^	7·9	35·6^a,b^	5·5	8·8^c^	3·2	20·8^b,c^	13·3
****22 : 4*n*-6 to 24 : 4*n*-6	61·2	5·4	46·2	7·3	32·9	4·9	4·0	2·5	17·8	14·1
Δ-5 desaturation	157·6^a^	15·8	85·0^a,b^	10·6	44·4^b,c^	6·9	31·7^b,c^	11·8	18·9^c^	10·2
Δ-6 desaturation	339·3^a^	51·3	225·7^a,b^	36·0	136·5^b,c^	10·4	96·6^b,c^	22·0	50·8^c^	33·3
****18 : 2*n*-6 to 18 : 3*n*-6	278·0^a^	45·8	179·5^a,b^	30·7	103·6^b,c^	5·6	92·7^b,c^	19·7	32·9^c^	19·2
****24 : 4*n*-6 to 24 : 5*n*-6	61·2	5·4	46·2	7·3	32·9	4·9	4·0	2·5	17·8	14·1
*n*-3 PUFA										
*β*-Oxidation	38·8	15·6	439·5	162·4	302·5	57·0	360·2	80·0	186·5	44·3
Elongation	58·4^b^	30·3	979·4^a^	208·9	141·0^b^	62·3	1221·1^a^	63·9	252·2^b^	46·4
****18 : 4*n*-3 to 20 : 4*n*-3	38·4^b^	18·2	414·9^a^	95·3	22·4^b^	2·6	509·3^a^	22·4	55·2^b^	21·2
****20 : 5*n*-3 to 22 : 5*n*-3	9·2	6·4	262·7	60·7	53·2	27·1	338·8	21·8	86·6	13·8
****22 : 5*n*-3 to 24 : 5*n*-3	9·4	5·8	230·1	51·9	65·4	33·1	310·5	20·4	105·7	11·4
Δ-5 desaturation	9·7^b^	8·1	323·4^a^	74·3	0·0^b^	0·0	409·7^a^	24·4	0·0^b^	0·0
Δ-6 desaturation	46·5^b^	24·4	842·9^a^	166·3	65·4^b^	33·1	992·5^a^	46·0	123·8^b^	29·5
****18 : 3*n*-3 to 18 : 4*n*-3	37·1^b^	18·6	612·8^a^	114·5	0·0^b^	0·0	681·9^a^	26·4	18·1^b^	18·1
****24 : 5*n*-3 to 24 : 6*n*-3	9·4	5·8	230·1	51·9	65·4	33·1	310·5	20·4	105·7	11·4
*n*-6 and *n*-3 PUFA										
Δ-5 desaturation	167·4^b^	21·5	408·4^a^	84·5	44·4^c^	6·9	441·4^a^	36·2	18·9^c^	10·2
Δ-6 desaturation	385·8^b^	67·1	1068·6^a^	201·3	201·9^b^	42·3	1089·1^a^	67·6	174·6^b^	62·8

LO, linseed oil-based diet; SO/LO, SO until day 60 and then LO from days 61–96. ^a,b,c^ Mean values within a row with unlike superscript letters were significantly different (Tukey’s (parametric, *α* 5 %) or Wilcoxon’s (non-parametric, *α* 1·69 %) *post hoc* tests on square root transformed values).

## Discussion

The aim of the present study was to evaluate the fatty acid bioconversion capacity of rainbow trout fry previously depleted in *n*-3 PUFA over a 60-d pre-experimental period and subsequently reverted to a diet rich in ALA or rich in EPA and DHA, for a 36-d experimental period. As controls, three other fish groups received SO, LO and FO throughout the 96-d feeding trial.

### Fish growth and proximate composition

A negative impact of SO was observed on fish growth performance in comparison to fish fed on LO or FO for 96 d. These results contrast with previous studies adding regular LA-rich sunflower oil or fish oil as only dietary lipid source in diets of Atlantic salmon^(^ ^44^ ^)^ and rainbow trout^(^ ^19^ ^)^ where no difference in fish growth and proximate composition between the two fish groups was reported. However, these studies were conducted on fish of a larger size and used fishmeal as the dietary protein source, which undoubtedly provided *n*-3 LC-PUFA to the diet, up to a level that might potentially meet the requirements for these health promoting nutrients. The fatty acid requirements of rainbow trout are 1 % ALA, 1 % LA and/or 0·5 % *n*-3 LC-PUFA in their diet (DM)^(^ ^5^ ^)^. The present lower growth of SO-fed fish was certainly due to the deficiency in essential ALA and *n*-3 LC-PUFA, as well as to an interconnected reduced feed intake. In contrast with the present results on SO fish, and in accordance with previous studies^(^ ^14^ ^,^ ^18^ ^,^ ^45^ ^,^ ^46^ ^)^, feeding LO for 96 d had no impact on fish growth. The replacement of SO by LO or FO for the 36-d experimental period significantly improved the growth of fish initially fed on SO. Indeed, the FE were higher in SO/LO and SO/FO fish groups as compared with the SO fish group and were similar to those observed in LO and FO fish groups, respectively. The present results demonstrate the rapid capacity of rainbow trout to cope with a change in dietary source. Turchini *et al*.^(^ ^47^ ^)^ previously reported enhanced growth, termed ‘lipo-compensatory growth’, of Murray cod fed a plant-derived oil diet and then a fish oil diet in comparison to fish fed a fish oil diet throughout. Similar observations were also reported in Atlantic salmon when shifted from rapeseed oil to a fish oil diet^(^ ^48^ ^)^ and for red seabream fed a soyabean oil diet for 3 months and then a fish oil diet for 32 d^(^ ^27^ ^)^.

### Fish fatty acid composition

At the end of the pre-experimental period (day 60), a high depletion in C18 *n*-3 PUFA and *n*-3 LC-PUFA was observed in fish of the SO treatment. This led to conclude on the efficiency of the pre-experimental period duration and in turn on the adequacy of the study design for evaluating the effects of the body *n*-3 PUFA depletion on the fish fatty acid bioconversion capacity. Interestingly, the *n*-3 PUFA depletion continued throughout the rest of the feeding trial for the SO treatment, following a decreasing exponential curve, as highlighted at days 70 and 96. Several studies^(^ ^14^ ^,^ ^19^ ^,^ ^22^ ^,^ ^23^ ^,^ ^44^ ^)^ have previously reported that the fatty acid composition of fish reflects that of the dietary lipid source. Similarly, in the present study, fish fed on the 18 : 1*n*-9-rich SO were the richest in 18 : 1*n*-9, whereas fish fed on LO presented a high ALA concentration while those fed on FO had the largest EPA and DHA concentrations. Certain discrepancies with the dietary fatty acid profile were evident in fish samples. For example, despite an absence of dietary *n*-6 LC-PUFA, these fatty acids were the highest in fish of the SO treatment, pointing towards an active *in vivo* metabolism. This result contrasts with previously published work on Atlantic salmon fed a 100 % LA-rich sunflower oil diet, where an increased 20 : 2*n*-6 concentration and decreased 20 : 4*n*-6 concentration were reported in comparison to fish fed a fish oil diet^(^ ^44^ ^)^. However, the present result is in line with the results of a study on rainbow trout fed a 100 % LA-rich sunflower oil diet in comparison to fish oil diet and linseed oil diet^(^ ^19^ ^)^. In similar fashion, fish fed on LO in the present study recorded the highest concentrations of *n*-3 PUFA fatty acid intermediates (18 : 4*n*-3, 20 : 3*n*-3 and 20 : 4*n*-3), despite being absent from the diet. The same observation was previously reported in rainbow trout fed a linseed oil diet for 112 d as compared with fish fed on sunflower oil diet or fish oil diet^(^ ^19^ ^)^. As previously observed by numerous studies, the present observations highlight, first, the relatively high capacity of rainbow trout to endogenously convert dietary LA and ALA into *n*-6 and *n*-3 LC-PUFA, respectively, and second, the modulation of the fish bioconversion capacity induced by the dietary lipid source^(^ ^9^ ^,^ ^14^ ^,^ ^17^ ^,^ ^19^ ^,^ ^20^ ^,^ ^22^ ^,^ ^31^ ^,^ ^49^ ^)^. Indeed, more bioconverted products were reported along the *n*-6 pathway in fish fed on SO considering that LA was one of major fatty acids present as substrate and that dietary ALA was almost absent, as previously observed in European sea bass^(^ ^31^ ^)^. Conversely, more bioconverted products of the *n*-3 pathway were observed in fish fed on LO as LA was present to a lesser extent than ALA and also considering the initial affinity of enzymes towards the *n*-3 PUFA as compared with the *n*-6 PUFA family^(^ ^14^ ^,^ ^15^ ^,^ ^49^ ^)^. A high recovery rate in *n*-3 PUFA was observed for SO/LO and SO/FO fish at the end of the 36-d experimental period. Indeed, SO/LO and SO/FO fish recovered a fatty acid profile with >80 % of the C18 *n*-3 PUFA and *n*-3 LC-PUFA values observed in fish fed on LO and FO, respectively, for 96 d. Interestingly, the transfer of Atlantic salmon previously fed a rapeseed oil diet for 50 weeks to a fish oil diet for 20 weeks also restored their EPA and DHA concentrations to 80 % of the levels found in fish fed on a fish oil diet for 70 weeks^(^ ^22^ ^)^. In European sea bass, 70 % recovery in EPA and DHA was reported in the flesh of fish fed a 40 % fish oil/60 % plant-derived oil blend for 64 weeks and then a finishing fish oil diet for a further 20 weeks, in comparison with fish fed on fish oil throughout^(^ ^26^ ^)^. However, two notable differences are apparent between both of these studies and the present one. Indeed, the results of the previous studies were based on fillet data from harvestable size fish whereas the present recovery rates are based on whole body fatty acid composition of fish from 20 to 50 g. Besides the recovery rates of 80 % observed at the end of the experimental period, the recovery rates in *n*-3 LC-PUFA were also reported for the 10th day of the period and achieved about 50 % for both the SO/LO and SO/FO fish groups. This means that the recovery in *n*-3 LC-PUFA was higher during the first 10 d of the 36-d experimental period than during the subsequent 26 d that followed. This observation corresponds to the well-established dilution kinetics following a decreasing exponential curve^(^ ^8^ ^,^ ^23^ ^,^ ^41^ ^,^ ^50^ ^)^. For example, this phenomenon was previously observed in Atlantic salmon fed a linseed oil diet for 40 weeks and then a fish oil diet for a further 24 weeks, where a DHA recovery rate of 83 % was observed by the end of the 24-week finishing period, while already reaching 79 % by the 16th week of the finishing period^(^ ^23^ ^)^. Interestingly, the DHA recovery rate was not slower and lower than that of the other *n*-3 LC-PUFA as the recovery rate values were similar on one hand on the short term (day 70) and on the other hand on the long term (day 96).

### *In vivo* fatty acid metabolism

The whole body fatty acid balance method clearly demonstrated the significantly increased apparent *in vivo* elongation and desaturation activities with regard to the *n*-3 biosynthesis pathway in fish fed on LO and the *n*-6 pathway in fish fed on SO. The high apparent *in vivo* bioconversion capacity of rainbow trout fed on plant-based diets is well established^(^ ^16^ ^,^ ^19^ ^,^ ^20^ ^)^ and is confirmed in the present study. In fish fed on LO, 25 % of the consumed ALA was being bioconverted into higher homologues on day 60 of the experiment, while this value reached 27 % on day 96. In comparison, 27 % of consumed ALA was also bioconverted in fish subjected to the SO/LO treatment from day 61 through day 96. In contrast with the present results, a previous study reported that only 12 % of consumed ALA was bioconverted in rainbow trout with an initial mean weight of approximately 90 g fed a linseed oil diet for 72 d, with the majority either being accumulated (58 %) or oxidised (30 %)^(^ ^20^ ^)^. However, that study used, on one hand, fish with a bigger size than ours, and, on the other hand, diets formulated with 7 % of fishmeal and therefore supplying fish with dietary EPA and DHA^(^ ^20^ ^)^.

At the end of the experimental period, no differences in apparent *in vivo* enzyme activity were observed along the *n*-3 pathway between the SO/LO and LO treatments. Moreover, no effects were observed on the 10th day of the experimental period. This indicates that the high *n*-3 PUFA depletion obtained with the SO treatment did not increase the apparent *in vivo* bioconversion of *n*-3 PUFA during the experimental period when ALA-rich linseed oil was present. It thus appears that the fish fatty acid composition has no importance, in contrast to the dietary fatty acid input, on the capacity of fish to convert ALA into *n*-3 LC-PUFA. Interestingly, the present study reported a significant impact of the *n*-3 PUFA depletion on the *n*-6 PUFA bioconversion capacity of SO/LO fish. Indeed, reduced apparent *in vivo* elongation, as well as apparent *in vivo* Δ-5 and Δ-6 desaturation activities along the *n*-6 pathway were observed in fish of the SO/LO treatment in comparison to those of the LO treatment. These decreased activities related to the *n*-6 pathway should point out that, in the case of fish previously depleted in *n*-3 PUFA, elongases and desaturases neglect the conversion of LA into *n*-6 LC-PUFA in the case of an ALA supply. Nevertheless, this did not correspond to increased apparent *in vivo* elongation or desaturation activities on the *n*-3 pathway and suggests that the effects are not always entirely predictable. In line with the results observed at the end of the 36-d experimental period, at the 10th day sampling point, the activities on the *n*-6 biosynthesis pathway appeared somewhat reduced in the SO/LO fish in comparison to the LO fish group. Recent studies have investigated the impact of *n*-3 PUFA-deprived diets on fish fatty acid metabolism and *n*-3 LC-PUFA deposition/ retention^(^ ^19^ ^,^ ^31^ ^,^ ^32^ ^)^. Francis *et al*.^(^ ^19^ ^)^ reported a modulatory effect on *n*-3 LC-PUFA deposition in rainbow trout fed a classic LA-rich sunflower oil diet and then a fish oil diet. The authors reported that the *n*-6 PUFA from the sunflower oil diet evoked a sparing of *n*-3 LC-PUFA from catabolism and resulted in higher *n*-3 LC-PUFA deposition in fish^(^ ^19^ ^)^. A similar sparing effect was also reported for sunshine bass (*Morone chrysops*×*Menticirrhus saxatilis*) fed a SFA-rich diet for which limited effects of fish oil replacement were reported on fillet fatty acid composition^(^ ^51^ ^,^ ^52^ ^)^. More precisely, sunshine bass fed a 50 % coconut oil diet and then a finishing fish oil diet recovered more effectively the *n*-3 LC-PUFA content observed for fish fed a fish oil diet throughout than fish fed three other diets formulated with 50 % grapeseed, linseed, or poultry oils for the grow-out period^(^ ^52^ ^)^. The authors concluded that dietary SFA appeared to be a preferential substrate for catabolism and induced an increased *n*-3 LC-PUFA deposition during the finishing period^(^ ^52^ ^)^. The present study reported no effect of the fish *n*-3 PUFA depletion on the apparent *in vivo* enzyme activity along the *n*-3 pathway in the SO/LO fish group, even on the 10th day of the experimental period. Further experiments should be set up to verify the absence of a transient metabolic adaptation in response to a previous shortage in dietary *n*-3 PUFA, for instance on the 2nd or 3rd days of the experimental period. The results of Hagar & Hazel^(^ ^53^ ^)^ support the validity of this suggestion by reporting that in rainbow trout acclimated at either 5 or 20°C and then transferred to the opposite temperature, an increase in hepatic Δ-6 desaturase activity within the first 3 d of temperature transfer before reverting to baseline values on the 6th day was observed. The whole body fatty acid balance method is nevertheless unsuitable for such short experimental periods of a few days and other evaluation tools should thus be used, such as gene expression and enzyme activity measurements at the tissue or cellular level^(^ ^31^ ^,^ ^54^ ^–^ ^58^ ^)^. These approaches should be implemented in further studies specifically focusing on tissues, such as liver and intestine, especially during the 1st day after dietary lipid replacement.

The present study is based on the *n*-3 PUFA depletion of fish with an initial mean weight of 0·7 g, which means fish that were previously fed on a standard diet for about 5 weeks. Complementary studies targeting the previously reported nutritional programming phenomenon^(^ ^31^ ^–^ ^33^ ^)^ may be performed. In such studies, the *n*-3 PUFA depletion starts at a much earlier stage, such as at the alevin stage and low *n*-3 LC-PUFA diets are used as first feeding and during a short period. For example, a 3-week early exposure of rainbow trout swim-up fry to a diet formulated with rapeseed oil, palm oil and linseed oil improved fish growth, feed intake and FE when the diet was used again 7 months later^(^ ^33^ ^)^. The lipid bioconversion capacity could also be improved by impacting broodstock. A recent study reported that feeding broodstock gilthead seabream with linseed oil induced long-term effects on the juvenile progeny fed a plant-based diet, as demonstrated by increased fish growth, FE and Δ-6 desaturase gene expression, as compared with juveniles from broodstock fed a fish oil diet^(^ ^59^ ^)^. In the present study, it was potentially tougher to highlight a difference of apparent *in vivo* enzyme activity than with other fish species, as rainbow trout possesses a high lipid bioconversion capacity. A similar experiment performed on another species possessing a reduced basal lipid bioconversion capacity might more readily highlight the potential stimulation of a *n*-3 PUFA depletion on the fatty acid bioconversion capacity. As examples, two previous studies on European sea bass reported increased Δ-6 desaturase gene expression in juveniles fed a *n*-3 LC-PUFA deficient diet when previously fed a *n*-3 LC-PUFA deficient larval diet, as compared with groups fed rich *n*-3 LC-PUFA larval diets^(^ ^31^ ^,^ ^32^ ^)^. In contrast, the lipid bioconversion capacity of common carp was not improved when fed a traditional cereal diet enriched with 1 % plant-derived oil for 180 d and then a finishing linseed oil diet or fish oil diet for 30 d^(^ ^24^ ^)^.

### Conclusions

The present study demonstrated that the initial high bioconversion capacity of rainbow trout to convert ALA into *n*-3 LC-PUFA was not modulated by a *n*-3 PUFA depletion of fish fatty acid composition through feeding for 60 d with a diet rich in sunflower oil. Indeed, the apparent *in vivo* enzyme activities related to that bioconversion remained stable along the *n*-3 fatty acid pathway. In contrast, the fish *n*-3 PUFA depletion negatively modulated the *n*-6 PUFA bioconversion capacity of fish in terms of reduced apparent *in vivo* elongation and desaturation enzyme activities, both on the 10th day and at the end of the 36-d experimental period. Further research on salmonids and other fish species is required to enhance the knowledge on fish fatty acid bioconversion metabolism and to improve fish bioconversion capacity through nutritional intervention strategies.
